# Feature Augmentation-Based Adaptive Neural Network Control for Quadrotors

**DOI:** 10.3390/s26031078

**Published:** 2026-02-06

**Authors:** Bang Song, Mengxing Huang

**Affiliations:** School of Information and Communication Engineering, Hainan University, Haikou 570228, China

**Keywords:** adaptive neural network (ANN), feature augmentation (FA), state predictor (SP), input-to-state stable (ISS), quadrotor

## Abstract

In this article, an adaptive neural network (ANN) controller based on feature augmentation (FA) is designed for quadrotors. The proposed controller consists of two components: a position sub-controller and an attitude sub-controller. We use the ANN to estimate unknown internal and external disturbance terms within quadrotors. To improve the learning accuracy of the ANN, we design an FA structure, which enables networks to more effectively learn the characteristics in the data. To increase the learning rate of the ANN, a state predictor (SP) is proposed to anticipate the state errors, which subsequently updates the learning rate of the ANN. Based on stability analysis, we prove that the closed-loop system is input-to-state stable (ISS). Finally, the effectiveness of our proposed control algorithm is demonstrated by comparing it with related control algorithms on both the MATLAB R2020a/Simulink simulation platform and a quadrotor experimental platform.

## 1. Introduction

With the widespread application of quadrotors, research in this field has garnered significant attention. At the same time, effective controller design is crucial for ensuring stable flight of quadrotors. For quadrotors, the unknown internal and external disturbance terms are the main factors affecting their airborne performance, including ground effect [1,2], air resistance [3,4], and others.

To effectively control quadrotor flight, several control methods have been proposed, including sliding mode control [5], fuzzy control [6], and proportional–integral–derivative (PID) control [7,8], among others. Owing to its simplicity and ease of tuning, PID control has been widely adopted in quadrotor flight applications. However, with the increasing complexity of operating scenarios, traditional PID control exhibits several inherent limitations [9,10], such as relying solely on current system states, lacking predictive capability for future dynamics, and being ineffective in estimating external disturbances, since it can only react to them. Although certain enhancements to PID control have been proposed, for example by integrating fuzzy logic [8], these approaches have not fully overcome the aforementioned drawbacks.

To address the effects of the unknown internal and external disturbance terms within quadrotors, a variety of efforts was applied to quadrotors. In [11], a disturbance observer was designed to handle external disturbances and ensure the finite-time stability for quadrotors. To deal with external disturbances of quadrotor formation in [12], a robust filter for each quadrotor was designed within the controller, which can restrain the effects of the disturbances. Based on the backstepping method, a robust adaptive control law was proposed in [13] to address external disturbances that are bounded. Similar to these works, neural networks (NNs) are commonly used to address disturbances caused by unknown internal nonlinear terms and external environmental factors [3,4,9,14], due to their excellent learning and approximation capabilities. To improve transient learning performance of NNs, prediction errors are used to update the NNs’ weights in [15] for uncertain nonlinear systems. To enhance the approximation capability of NNs, fully connected recurrent neural networks (RNNs) with feedback to the input layer and hidden layer are proposed to combine with the sliding mode control for quadrotors [16]. In [17], reinforcement learning, constructed on the basis of NNs, aims to approximate the solution of the Hamilton–Jacobi–Bellman equation. During the transition process in [18], an $L_{1}$ NN adaptive controller associated with PID is proposed to address fast time-varying disturbances, which improves the compensation ability and guarantees security. In these studies, while NNs emerge as promising solutions for the discussed issues, their implementation has so far been confined to simulation environments, with limited real-world applications, especially in the design of quadrotor controllers based on NNs.

Furthermore, with the upgrading of hardware equipment, there are studies exploring the application of NNs in real-world scenarios. To address issues arising from unknown time-varying disturbances, a NN-based controller with online weight determination in [3] was designed for the quadrotor. Similar to the approach in [18], NNs were also integrated with the PID controller in [9]. However, the key distinction lies in the experimental validation of the controller in [9]. In [19], a discrete-time adaptive dynamic surface control with radial basis function NNs was proposed for the quadrotor to achieve satisfactory tracking performance in experimental implementation.

Based on the above discussion, the basic structure of NNs can be mainly classified into two types according to their learning methods: feed-forward NNs, such as radial basis function (RBF) NNs [3,14,20], and feedback NNs, such as recurrent neural networks (RNNs) [16,21] and echo state networks (ESNs) [22]. In engineering applications, especially in quadrotor flight control, feed-forward NNs are often preferred to estimate the disturbances caused by unknown internal nonlinear terms and the external environment. This preference arises from the constraints of hardware size and computational capabilities of quadrotors.

**Figure 1:**
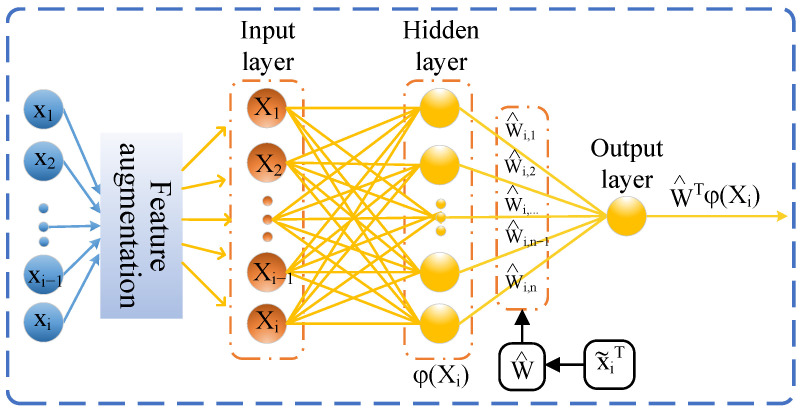
Architecture of ANN based on FA.

Building on the aforementioned work [3,4,9,14], we made a structural enhancement to feed-forward NNs by incorporating a feature augmentation (FA) layer, as shown in Figure 1. This addition aims to improve the approximation of unknown internal and external disturbance terms in quadrotors.

This paper presents the design and implementation of an ANN controller using the backstepping method, augmented with the FA, which can enable the ANN to more effectively learn and capture the characteristics of the input data. Additionally, we improve the learning efficiency of the ANN by updating the network weights based on errors predicted by the state predictor (SP), rather than directly using the state errors. We verified the controller through both simulations and real-world experiments. The main contributions of this paper are as follows:

1. Unlike the NN-based control techniques proposed in [3,4,9,14,16,23], as shown in Figure 1, our approach enhances characterization of the NNs inputs. By adding the FA layer to the NN structure, we improve the learning accuracy of NNs to approximate and compensate for unknown internal and external disturbance terms in quadrotors.
2. Compared with the existing literature [3,4,9,16,19,23], in addition to adding the FA layer, we design an SP to improve the NNs’ approximation speed to the unknown disturbance terms. This predictor estimates the NN inputs and updates the networks’ weights based on the estimation errors, rather than directly using the state errors.
3. Unlike previous studies such as [5,7], which validated NNs control only through simulations, our work includes both MATLAB/Simulink and real quadrotor experiments. We compared our ANN controller, based on the FA with SP against traditional PID and RBF NNs with SP controllers. The simulation and experiment results verify the effectiveness of our controller, which ensures the input-to-state stability (ISS) of the quadrotor system using the Lyapunov theory.

## 2. Preliminaries

### 2.1. Notations

Throughout this paper, bold symbols such as p, v, etc., indicate variables defined in multidimensional space. ∥·∥ denotes the Euclidean norm of a vector, ${∥\cdot ∥}_{F}$ denotes the Frobenius norm of a matrix. The symbol $\hat{\left(\cdot \right)}$ represents the estimated value of (·), and $\tilde{\left(\cdot \right)}=\hat{\left(\cdot \right)}-\left(\cdot \right)$. The operator ∘ denotes the Hadamard product, and diag{…} denotes a diagonal matrix. $\lambda_{\min}$ and $\lambda_{\max}$ represent the minimum and maximum eigenvalues of a square matrix, respectively. The notation A:=B represents that *B* is defined as *A*, and $SO(3):=\{X\in R^{3\times 3}:XX^{T}=X^{T}X=I,\det\left(X\right)=1\}$ is the special orthogonal group. $e_{1}:={\left[1,0,0\right]}^{T}$, $e_{2}:={\left[0,1,0\right]}^{T}$, and $e_{3}:={\left[0,0,1\right]}^{T}$ are defined as unit vectors.

### 2.2. Quadrotor Model

**Figure 2:**
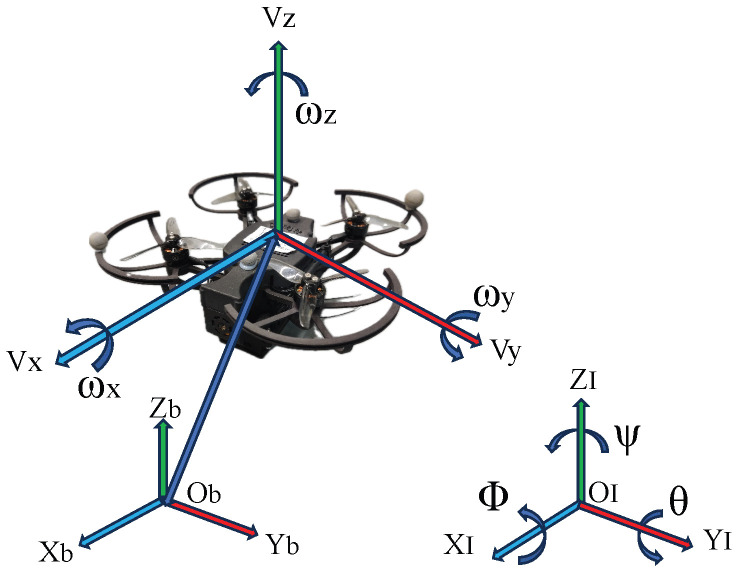
A sketch of the quadrotor.

In this paper, based on the Newton–Euler equations, we use two coordinate systems to better describe the attitude of the quadrotor: the inertial frame {I} and the body frame {B}, as shown in Figure 2. The origin of {I} coincides with the center of {B}.

The classic kinematic model, which contains both translation and rotation information, is described as:

(1) $$\begin{array}{cc} \dot{p} & =v, \end{array}$$

(2) $$\begin{array}{cc} \dot{\Theta } & =R_{\omega }\omega , \end{array}$$

where $p\in R^{3\times 1}$ and $v\in R^{3\times 1}$ represent the position and linear velocity of the quadrotor, respectively. In inertial frame {I}, $\Theta ={\left[\phi ,\theta ,\psi \right]}^{T}$ denotes the rotation angle for quadrotor attidude change and $\omega \in R^{3\times 1}$ represents the angular velocity. $R_{\omega }$ is a spatial variation matrix with Θ, which is used to show the conversion relationship between the rate of attitude change in {I} and the angular velocity in {B} of the quadrotor. Based on Newton’s second law, as discussed in [24], the dynamic model is described as follows:

(3) $$\begin{array}{cc} \dot{v} & =m^{-1}f+ge_{3}+\Delta_{d}, \end{array}$$

(4) $$\begin{array}{cc} \dot{\omega } & =J^{-1}\left(-S\left(\omega \right)J\omega +\tau \right), \end{array}$$

where *m* and *g* represent the mass and gravitational acceleration of the quadrotor, respectively. The moment of inertia $J\in R^{3\times 3}$ is a symmetric positive definite matrix, and the function S(ω) satisfying S(ω)J=ωJ, is the skew-symmetric matrix. $\Delta_{d}\in R^{3\times 1}$ represents unknown internal and external disturbance terms for the quadrotor. $f\in R^{3\times 1}$ denotes the thrust, and T∈R is the combined pull force generated by the propellers of the quadrotor [25]. The relationship between f and *T* is given by $f:=-TRe_{3}$, where R∈SO(3) represents the rotation matrix mapping the vectors from the body frame {B} to the inertial frame {I}. $\tau \in R^{3\times 1}$ indicates the torque generated by the propellers of the quadrotor.

### 2.3. NNs Based on FA Formulation

Any smooth function f(·) with f(0)=0 can be defined by RBF NNs [4], which has great capabilities in function approximation with arbitrary precision. However, when the number of learning samples is insufficient or the sample dimensions are low, its learning and approximation effectiveness decreases [26]. Considering the dimensional characteristics of the quadrotor model, we added the FA layer, as shown in Figure 1, to the traditional feed-forward NNs. This modification mainly includes the Hadamard product [27,28,29] and a weight matrix to improves the NNs’ ability to approximate the unknown disturbance terms in quadrotors. The RBF NNs based on the FA can be expressed as:

(5) $$f_{\mathrm{FA}}\left(x\right)=W^{T}\varphi \left(W_{\mathrm{FA}}\left(x\circ x\right)\right)+\epsilon \left(\cdot \right),$$

where $x\in R^{n\times 1}$ is the input vector for NNs, $W_{\mathrm{FA}}\in R^{n\times n}$ is the weight matrix in the FA layer, $\epsilon \left(\cdot \right)\in R^{n\times 1}$ represents the network approximation error, and $W\in R^{d\times 3}$ with *d* being the number of neurons, is the ideal weight vector of the NNs, $\tilde{W}=\hat{W}-W$. The function $\varphi \left(\cdot \right)\in R^{d\times 1}$ represents the activation function.

**Remark** **1.** *All network weights, including those in the FA layer and output layer, are initialized to small random values (e.g., uniformly in [−0.01, 0.01]). This ensures symmetry breaking and prevents saturation, enabling effective online adaptation. The closed-loop performance is robust to the precise initial values due to the adaptive nature of the learning process.*

### 2.4. Control Objective

In this paper, a robust controller is developed for quadrotors, consisting of ANN based on the FA with SP. The controller is designed for the classic kinematic model (1), (2) and the dynamic model (3), (4). The design of the controller aims to achieve the following objectives:

1. With the control laws designed in this paper, validated through simulation and experiments, the quadrotor can quickly track the desired trajectory $p_{d}\in R^{3\times 1}$.
2. We design ANN based on FA with an SP to estimate and compensate for unknown internal and external disturbance terms $\Delta_{d}$ quickly and efficiently, compared to traditional RBF NNs.
3. The system errors converge to a small bounded area, and the closed system is proven to be ISS.

**Assumption** **1.** *The desired trajectory $p_{d}$ is twice differentiable and bounded.*

**Assumption** **2.** *For the quadrotor, |ϕ|<π/2 and |θ|<π/2.*

**Assumption** **3.** *The influence of unknown disturbance terms on the quadrotor attitude is relatively small and can be neglected. The unknown internal and external disturbance terms $\Delta_{d}$ are bounded, nonlinear, and unknown, satisfying $∥\Delta_{d}∥\leq {\bar{\Delta }}_{d}$, where ${\bar{\Delta }}_{d}\in R^{+}$.*

## 3. Controller Design

**Figure 3:**
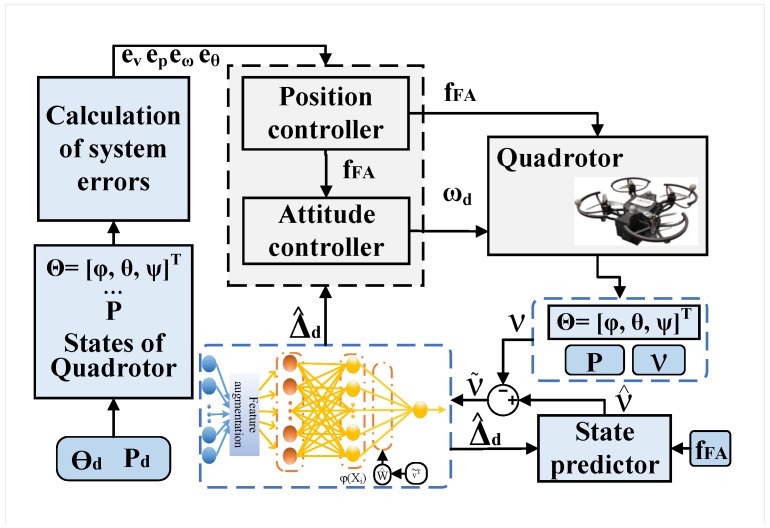
Flowchart of ANN controller based on FA with SP for quadrotors.

As shown in Figure 3, a flowchart depicting the control process, an ANN controller based on the FA with an SP is designed for quadrotors in this section. The controller includes a position sub-controller and an attitude sub-controller to achieve the control objectives outlined in Section 2.4. This design aims to enhance the quadrotor’s stability and anti-interference performance in the presence of unknown internal and external disturbance terms $\Delta_{d}$.

### 3.1. Position Sub-Controller

To enable the quadrotor to track a desired trajectory $p_{d}$, we define the position error $e_{p}$ as follows:

(6) $$e_{p}=p-p_{d}.$$

Using (1) and (6), we take the derivative of $e_{p}$ and obtain:

(7) $${\dot{e}}_{p}=v-{\dot{p}}_{d}.$$

Applying the backstepping method, the ideal velocity $v_{d}$ of the quadrotor can be designed as:

(8) $$v_{d}={\dot{p}}_{d}-K_{p}e_{p},$$

where $K_{p}\in R^{3\times 3}$ is a diagonal matrix representing the control gains. Substituting (8) into (7), we obtain ${\dot{e}}_{p}=-K_{p}e_{p}$. Then, the velocity error $e_{v}$ of the quadrotor is defined as:

(9) $$e_{v}=K_{p}e_{p}+v-{\dot{p}}_{d}.$$

Combining with (3), we can calculate the time derivative of $e_{v}$ as:

(10) $${\dot{e}}_{v}=\dot{v}-{\dot{v}}_{d}=m^{-1}f+ge_{3}-{\dot{v}}_{d}+\Delta_{d}.$$

Based on (10), the desired control law f is designed as:

(11) $$f=-mge_{3}+m{\dot{v}}_{d}-K_{v}me_{v}-m\Delta_{d},$$

where $K_{v}\in R^{3\times 3}$ is a diagonal matrix used to adjust gains. Substituting (11) into (10), we get ${\dot{e}}_{v}=-K_{v}e_{v}$. Then, the ANN based on feature augmentation $f_{\mathrm{FA}}$ as designed in Section 2.3 are designed to approximate the unknown terms $\Delta_{d}$ as:

(12) $${\hat{\Delta }}_{d}={\hat{W}}^{T}\varphi \left(W_{\mathrm{FA}}\left(\Xi \circ \Xi \right)\right),$$

where $\Xi ={\left[v^{T}\left(t\right),v^{T}\left(t-t_{d}\right),e_{v}^{T},{\left(ge_{3}\right)}^{T}\right]}^{T}\in R^{12}$ is the input vector of the ANN as shown in Figure 1 with $t_{d}$ is the sampling time, and ${\hat{\Delta }}_{d}$ is the estimate of $\Delta_{d}$.

Combining (11) and (12), we design the ANN controller $f_{\mathrm{FA}}={\left[f_{\mathrm{FAx}},f_{\mathrm{FAy}},f_{\mathrm{FAz}}\right]}^{T}$ as follows:

(13) $$f_{\mathrm{FA}}=-mge_{3}-m{\hat{W}}^{T}\varphi \left(W_{\mathrm{FA}}\left(\Xi \circ \Xi \right)\right)+m{\dot{v}}_{d}-K_{v}me_{v}.$$

In order to improve the ANN approximation efficiency and reduce overshooting, we design an SP as follows:

(14) $$\dot{\hat{v}}={\hat{W}}^{T}\varphi \left(W_{\mathrm{FA}}\left(\Xi \circ \Xi \right)\right)+m^{-1}f_{\mathrm{FA}}+ge_{3}-K_{FA}\tilde{v},$$

where $K_{\mathrm{FA}}\in R^{3\times 3}$ is a tuning parameter matrix for the SP. The update law for the weights $\hat{W}$ of the ANN is designed as:

(15) $$\dot{\hat{W}}=\eta \left(-\varphi \left(W_{\mathrm{FA}}\left(\Xi \circ \Xi \right)\right){\tilde{v}}^{T}-\sigma \hat{W}\right),$$

where η and σ are positive constants.

**Remark** **2.** *The state predictor (SP) enhances disturbance estimation accuracy by using real-time prediction errors to adaptively update the ANN weights. This mechanism allows the controller to quickly adapt to rapidly changing disturbances, while the regularization term ensures stability. Including both current and past states in the input further improves performance under dynamic conditions.*

### 3.2. Attitude Sub-Controller

We define the rotation angle error of the quadrotor as:

(16) $$e_{\Theta }=\Theta -\Theta_{d},$$

as designed in [12], the desired attitude angles $\Theta_{d}={\left[\phi_{d},\theta_{d},\psi_{d}\right]}^{T}$ for the quadrotor are determined as follows:

(17) $$\begin{array}{c} \left\{\begin{array}{c} \phi_{d}:=\sin^{-1}\left(f_{\mathrm{FAx}}\sin\psi /T-f_{\mathrm{FAy}}\cos\psi /T\right)\\ \theta_{d}:=\tan^{-1}\left(f_{\mathrm{FAx}}\cos\psi /f_{\mathrm{FAz}}+f_{\mathrm{FAy}}\sin\psi /f_{\mathrm{FAz}}\right)\\ \psi_{d}:=0. \end{array}\right. \end{array}$$

By taking the derivative of $e_{\Theta }$ based on (2) and (16), we obtain:

(18) $${\dot{e}}_{\Theta }=R_{\omega }\omega -{\dot{\Theta }}_{d}.$$

Then, $\omega_{d}$ can be obtained by applying the backstepping method with (2), (16), and (18) as follows:

(19) $$\omega_{d}=R_{\omega }^{-1}{\dot{\Theta }}_{d}-e_{\Theta }R_{\omega }^{-1}e_{\Theta },$$

where $e_{\Theta }\in R^{3\times 3}$ is a positive diagonal gain matrix. Substituting (19) into (18), we get ${\dot{e}}_{\Theta }=-e_{\Theta }e_{\Theta }$. The angular velocity error $e_{\omega }$ is defined as:

(20) $$e_{\omega }=\omega +e_{\Theta }R_{\omega }^{-1}e_{\Theta }-R_{\omega }^{-1}{\dot{\Theta }}_{d}.$$

Then, we get the derivative of $e_{\omega }$ as:

(21) $${\dot{e}}_{\omega }=J^{-1}\tau -J^{-1}S\left(\omega \right)J\omega -{\dot{\omega }}_{d}.$$

Similar to the solution process of Equation (11), the desired torque input is designed as follows:

(22) $$\tau =J{\dot{\omega }}_{d}+S\left(\omega \right)J\omega -K_{\omega }Je_{\omega },$$

where $K_{\omega }\in R^{3\times 3}$ is a positive diagonal gain matrix. Substituting (22) into (21), we get ${\dot{e}}_{\omega }=-K_{\omega }e_{\omega }$.

**Remark** **3.** *We propose an adaptive neural network controller that integrates a Feature Augmentation (FA) layer, which improves the network’s ability to approximate disturbances more accurately. This is a key innovation, as previous neural network-based methods, such as RBF networks, struggle with low-dimensional input spaces and slow learning rates in high-dimensional tasks like quadrotor control. Furthermore, we introduce a state predictor (SP) to estimate state errors in advance, allowing the controller to dynamically adjust the learning rate of the neural network. This proactive learning mechanism enables faster adaptation to disturbances, which is not found in previous methods that rely solely on state error feedback for updating weights.*

**Remark** **4.** *The mapping from the controller outputs $f_{FA}$ and $\omega_{d}$ to the physical inputs of the quadrotor is completed as follows: The thrust command T is obtained by projecting $f_{FA}$ onto the body z-axis. The torque command **τ** is calculated using a backstepping-based attitude controller to ensure **ω** tracks $\omega_{d}$.*

## 4. Stability Analysis

Based on the controller design in the previous section, this section analyzes the stability of the closed-loop system for the quadrotor through the error dynamics, which can be expressed as follows:

(23) $$\begin{array}{c} \left\{\begin{array}{c} {\dot{e}}_{p}=-K_{p}e_{p}\\ {\dot{e}}_{v}=-K_{v}e_{v}\\ {\dot{e}}_{\Theta }=-K_{\Theta }e_{\Theta }\\ {\dot{e}}_{\omega }=-K_{\omega }e_{\omega }\\ \dot{\tilde{v}}={\tilde{W}}^{T}\varphi \left(W_{FA}\left(\Xi \circ \Xi \right)\right)-K_{FA}\tilde{v}-\epsilon \left(\cdot \right)\\ \dot{\tilde{W}}=\eta \left(-\varphi \left(W_{FA}\left(\Xi \circ \Xi \right)\right){\tilde{v}}^{T}-\sigma \hat{W}\right). \end{array}\right. \end{array}$$

**Theorem** **1.** *For the quadrotor system described by *(1)*–*(4)*, along with the control laws designed in *(8)*, *(11)*, *(19)* and *(22)*, and the adaptive update laws of the NNs based on FA with SP established in *(14)* and *(15)*, the closed-loop system *(23)* is input-to-state stable (ISS).*

**Proof.**  Define a Lyapunov function $V_{cl}$ for (23) as:

(24) $$\begin{array}{c} V_{cl}\;=\;\frac{1}{2}\left[e_{p}^{T}e_{p}\;+\;e_{v}^{T}e_{v}\;+\;e_{\Theta }^{T}e_{\Theta }\;+\;e_{\omega }^{T}e_{\omega }\;+\;{\tilde{v}}^{T}\tilde{v}\;+\;\frac{1}{\eta }tr\left({\tilde{W}}^{T}\;\tilde{W}\right)\right]. \end{array}$$

Using (23) to take the derivative of $V_{cl}$, which can be expressed as follows:

(25) $$\begin{array}{c} \begin{array}{cc} {\dot{V}}_{cl} & =e_{p}^{T}{\dot{e}}_{p}+e_{v}^{T}{\dot{e}}_{v}+e_{\Theta }^{T}{\dot{e}}_{\Theta }+e_{\omega }^{T}{\dot{e}}_{\omega }+{\tilde{v}}^{T}\dot{\tilde{v}}+\frac{1}{\eta }tr\left({\tilde{W}}^{T}\dot{\tilde{W}}\right)\\ \; & =-K_{p}\left|\right|e_{p}{\left|\right|}^{2}-K_{v}\left|\right|e_{v}{\left|\right|}^{2}-K_{\Theta }\left|\right|e_{\Theta }{\left|\right|}^{2}-K_{\omega }\left|\right|e_{\omega }{\left|\right|}^{2}\\ & -K_{FA}\left|\right|\tilde{v}{\left|\right|}^{2}-\sigma ||\tilde{W}{\left|\right|}_{F}^{2}-{\tilde{v}}^{T}\epsilon \left(\cdot \right)-\sigma tr\left({\tilde{W}}^{T}W\right). \end{array} \end{array}$$

By applying mathematical methods such as Young’s inequality to the deflationary transformation of (25), we obtain the following inequalities:

(26) $$\begin{array}{c} \begin{array}{cc} {\dot{V}}_{cl} & \leq -\lambda_{\min}\left(K_{p}\right)\left|\right|e_{p}{\left|\right|}^{2}-\lambda_{\min}\left(K_{v}\right)\left|\right|e_{v}{\left|\right|}^{2}\\ & -\lambda_{\min}\left(K_{\Theta }\right)\left|\right|e_{\Theta }{\left|\right|}^{2}-\;\lambda_{\min}\left(K_{\omega }\right)\left|\right|e_{\omega }{\left|\right|}^{2}\\ & -\lambda_{\min}\left(K_{FA}\right)\left|\right|\tilde{v}{\left|\right|}^{2}-\sigma ||\tilde{W}{\left|\right|}^{2}+\left|\right|\tilde{v}||||\epsilon \left(\cdot \right)\left|\right|\\ & +\sigma ||\tilde{W}{\left|\right|}_{F}{\left||W|\right|}_{F}. \end{array} \end{array}$$

To further simplify (26), we define:

(27) $$\begin{array}{c} E_{cl}={\left[\left|\right|e_{p}\left||,|\right|e_{v}\left||,|\right|e_{\Theta }\left||,|\right|e_{\omega }\left||,|\right|\tilde{v}\left||,|\right|\tilde{W}{\left|\right|}_{F}\right]}^{T}, \end{array}$$

and

(28) $$\begin{array}{c} \begin{array}{cc} c_{cl}= & \min\{\lambda_{\min}\left(K_{p}\right),\lambda_{\min}\left(K_{v}\right),\lambda_{\min}\left(K_{\Theta }\right),\lambda_{\min}\left(K_{\omega }\right),\\ & \lambda_{\min}\left(K_{FA}\right),\sigma \}. \end{array} \end{array}$$

Then we can rewrite ${\dot{V}}_{cl}$ as:

(29) $$\begin{array}{c} \begin{array}{cc} {\dot{V}}_{cl} & \leq -c_{cl}\left|\right|E_{cl}{\left|\right|}^{2}+\left|\right|\tilde{v}\left||||\epsilon \left(\cdot \right)|\right|+\sigma ||\tilde{W}{\left|\right|}_{F}{\left||W|\right|}_{F}\\ & \leq -c_{cl}\left|\right|E_{cl}{\left|\right|}^{2}+\left|\right|H_{cl}\left|||\right|E_{cl}\left|\right|, \end{array} \end{array}$$

where $H_{cl}={\left[0,0,0,0,||\epsilon \left(\cdot \right)||,\sigma ||\tilde{W}{\left|\right|}_{F}\right]}^{T}$. $\forall ||E_{cl}\left|\right|\geq 2||H_{cl}\left|\right|/c_{cl}$ makes ${\dot{V}}_{cl}\leq -c_{cl}\left|\right|E_{cl}{\left|\right|}^{2}/2$, which makes the system (23) ISS. In order to further analyze the ISS properties of the closed-loop system, we introduce a KL function and a series K functions. The stability analysis can be conducted under the following two scenarios: (a) The initial states without external disturbances; (b) Persistent disturbances.

**(a) The Initial States without External Disturbances:** Without external disturbances, the decay of the system states can be described by the KL function. We define the KL functions as $\zeta \left(o^{*},t\right)=o^{*}\exp\left(-\bar{\alpha }t\right)$, where $\bar{\alpha }$ represents the decay rate of the function. Assuming that there is no disturbance input, i.e., ϵ(·)=0 and $\tilde{W}=0$, then:
  (30) $${\dot{V}}_{cl}\leq -c_{cl}\left|\right|E_{cl}{\left|\right|}^{2}.$$

We integrate ${\dot{V}}_{cl}$ to obtain:

(31) $$\int_{0}^{t}{\dot{V}}_{cl}\left(y\right)dy\leq \int_{0}^{t}-c_{cl}\left|\right|E_{cl}\left(y\right){\left|\right|}^{2}dy.$$

Then, from (31), we can get:

(32) $$V_{cl}\left(E_{cl}\left(t\right)\right)-V_{cl}\left(E_{cl}\left(0\right)\right)\leq -c_{cl}\int_{0}^{t}\left|\right|E_{cl}\left(y\right){\left|\right|}^{2}dy.$$

Since $V_{cl}\left(E_{cl}\left(t\right)\right)\leq 0$, we get:

(33) $$V_{cl}\left(E_{cl}\left(t\right)\right)\leq V_{cl}\left(E_{cl}\left(0\right)\right)-c_{cl}\int_{0}^{t}\left|\right|E_{cl}\left(y\right){\left|\right|}^{2}dy.$$

Further simplification yields:

(34) $$V_{cl}\left(E_{cl}\left(t\right)\right)\leq V_{cl}\left(E_{cl}\left(0\right)\right).$$

Since the Lyapunov fuction $V_{cl}\left(E_{cl}\left(t\right)\right)\leq 0$, there exist constants $k_{1}$ and $k_{2}$ such that: $k_{1}\left|\right|E_{cl}{\left(t\right)||}^{2}\leq V_{cl}\left(E_{cl}\left(t\right)\right)\leq k_{2}\left|\right|E_{cl}\left(t\right){\left|\right|}^{2}$. Utilizing the upper and lower bound properties of the Lyapunov function, we obtain:

(35) $$k_{1}\left|\right|E_{cl}{\left(t\right)||}^{2}\leq V_{cl}\left(E_{cl}\left(t\right)\right)\leq V_{cl}\left(E_{cl}\left(0\right)\right)\leq k_{2}\left|\right|E_{cl}\left(0\right){\left|\right|}^{2}.$$

From (35), we get:

(36) $$\left|\right|E_{cl}\left(t\right)||\leq \sqrt{\frac{k_{2}}{k_{1}}}\left|\right|E_{cl}\left(0\right)\left|\right|.$$

Combined (36) with the properties of KL functions, we obtain: $V_{cl}\left(E_{cl}\left(t\right)\right)\leq V_{cl}\left(E_{cl}\left(0\right)\right)\exp\left(-c_{cl}t\right)$. Thus, we can derive the following equation:

(37) $$\left|\right|E_{cl}\left(t\right)||\leq \sqrt{\frac{k_{2}}{k_{1}}}\left|\right|E_{cl}\left(0\right)\left|\right|\exp\left(\frac{-c_{cl}t}{2}\right).$$

Equation (37) describes the initial state of the system exhibiting exponential decay over time, indicating that the state of the system will gradually converge to zero.

**(b) Persistent Disturbances:** In the presence of perturbing inputs, the following equation can be derived from (29):
  (38) $$\begin{array}{c} \begin{array}{c} {\dot{V}}_{cl}\leq -\frac{c_{cl}}{2}\left|\right|E_{cl}{\left|\right|}^{2}-\left|\right|E_{cl}\left|\right|\left(\frac{c_{cl}}{2}\left|\right|E_{cl}\left|\right|-\left|\right|H_{cl}\left|\right|\right). \end{array} \end{array}$$

Now, we will analyze the term $\left(\frac{c_{cl}}{2}\left|\right|E_{cl}\left|\right|-\left|\right|H_{cl}\left|\right|\right)$. When $\left|\right|E_{cl}\left|\right|\geq \frac{2||H_{cl}\left|\right|}{c_{cl}}$, we have $\frac{c_{cl}}{2}\left|\right|E_{cl}\left|\right|-\left|\right|H_{cl}\left|\right|\geq 0$, which implies:

(39) $${\dot{V}}_{cl}\leq -c_{cl}\left|\right|E_{cl}{\left|\right|}^{2}.$$

From (39), we can conclude that when $\left|\right|E_{cl}\left|\right|\geq \frac{2||H_{cl}\left|\right|}{c_{cl}}$, the state of the system decays exponentially and stabilizes. When $\left|\right|E_{cl}\left|\right|\leq \frac{2||H_{cl}\left|\right|}{c_{cl}}$, we can rewrite $\left|\right|H_{cl}\left|\right|$ as:

(40) $$\left|\right|H_{cl}\left|\right|=\sqrt{{\left||\epsilon \left(\cdot \right)|\right|}^{2}+\left(\sigma |\right|\tilde{W}{\left|\right|}_{F}^{2})}.$$

We define two K-functions $k_{\epsilon }\left(r\right)=\frac{2r_{1}}{c_{cl}}$ and $k_{w}\left(r\right)=\frac{2\sigma r_{2}}{c_{cl}}$, respectively, where $r_{1}=\left||\epsilon \left(\cdot \right)|\right|$ and $r_{2}=\left|\right|\tilde{W}{\left|\right|}_{F}$. When the system is stable, we expect $\left|\right|E_{cl}\left|\right|\leq \frac{2}{c_{cl}}\left|\right|H_{cl}\left|\right|$. According to (40), we obtain:

(41) $$\left|\right|E_{cl}\left|\right|\leq \sqrt{{\left(\frac{2||\epsilon (\cdot )||}{c_{cl}}\right)}^{2}+(\frac{2\sigma ||\tilde{W}{\left|\right|}_{F})}{c_{cl}}}{)}^{2}.$$

By applying the Minkowski inequality, we get:

(42) $$\left|\right|E_{cl}\left|\right|\leq \frac{2||\epsilon (\cdot )||}{c_{cl}}+\frac{2\sigma ||\tilde{W}{\left|\right|}_{F})}{c_{cl}}.$$

According to the properties of the K functions $k_{\epsilon }\left(||\epsilon \left(\cdot \right)||\right)$ and $k_{w}\left(|\right|\tilde{W}{\left|\right|}_{F})$, the upper bound of the system can be expressed as:

(43) $$\left|\right|E_{cl}\left(t\right)||\leq k_{\epsilon }\left(||\epsilon \left(\cdot \right)|\right|+k_{w}\left(|\right|\tilde{W}{\left|\right|}_{F}).$$

Therefore, we can conclude that when the system is affected by perturbing inputs, the effects are bounded. Combining the two scenarios, we obtain:

(44) $$\begin{array}{c} \begin{array}{cc} \left|\right|E_{cl}\left(t\right)\left|\right| & \leq \max\{\sqrt{\frac{k_{2}}{k_{1}}}\left|\right|E_{cl}\left(0\right)\left|\right|\exp\left(\frac{-c_{cl}t}{2}\right),\\ & k_{\epsilon }\left(||\epsilon \left(\cdot \right)||\right)+k_{w}\left(|\right|\tilde{W}{\left|\right|}_{F})\}. \end{array} \end{array}$$

Thus, all error signals within the quadrotor system are uniformly bounded. □

**Theorem** **2.** *For the disturbance function $\Delta_{d}\left(\Xi \right)$ of the nonlinear system, if the neural network employs feature augmentation (FA) by including quadratic terms of the input, then the approximation error of the FA-based NN, $\left|\right|E_{FA}\left|\right|$, is strictly less than that of the conventional feed-forward NN, $\left|\right|E_{NN}\left|\right|$, for any Ξ≠0, i.e., $\left|\right|E_{NN}\left||>|\right|E_{FA}\left|\right|$.*

**Proof.**  By Taylor’s theorem, the disturbance function $\Delta_{d}\left(\Xi \right)$ can be expanded around Ξ=0 as:

(45) $$\Delta_{d}\left(\Xi \right)=\Delta_{d}\left(0\right)+\nabla \Delta_{d}{|}_{0}\Xi +\frac{1}{2}\Xi^{T}H_{d}\Xi +{o(∥\Xi ∥}^{3}),$$

where $\nabla \Delta_{d}{|}_{0}$ is the gradient and $H_{d}$ is the Hessian.

**(1) Conventional Feed-forward NN:**

$$\Delta_{d}\left(\Xi \right)\approx {\hat{W}}_{NN}^{T}\varphi \left(\Xi \right),$$

where φ(·) is the NN activation. The approximation error is:

(46) $$E_{NN}=\Delta_{d}\left(0\right)+\nabla \Delta_{d}{|}_{0}\Xi +\frac{1}{2}\Xi^{T}H_{d}\Xi -{\hat{W}}_{NN}^{T}\varphi \left(\Xi \right).$$

**(2) FA-based NN (with quadratic input terms):**

$$\Delta_{d}\left(\Xi \right)\approx {\hat{W}}_{FA}^{T}\varphi \left(\left[\Xi ;\Xi \circ \Xi \right]\right),$$

where [Ξ;Ξ∘Ξ] denotes concatenation of Ξ and its element-wise square. The approximation error is:

(47) $$\begin{array}{cc} E_{FA}=\Delta_{d}\left(0\right)+\nabla \Delta_{d}{|}_{0}\Xi & +\frac{1}{2}\Xi^{T}H_{d}\Xi \\ \; & -{\hat{W}}_{FA}^{T}\varphi \left(\left[\Xi ;\Xi \circ \Xi \right]\right). \end{array}$$

Since the FA-based NN explicitly includes both linear and quadratic input terms, it can better match the Taylor expansion up to the second-order, while the conventional NN can only fit the constant and linear terms (or requires more hidden units to approximate quadratic terms implicitly).

Therefore, for any Ξ≠0, the norm of the approximation error for the FA-based NN is strictly less than that of the standard NN:

$$\left|\right|E_{NN}\left||>|\right|E_{FA}\left|\right|,$$

which completes the proof. □

## 5. Simulation Examples

In this section, we design a parameterised reference trajectory p(ν) = [0.6sin(0.4ν),${0.7\cos\left(0.3\nu \right),0.1\nu ]}^{T}\left(m\right)$ with the variable ν(t), where $\ddot{\nu }\left(t\right)=\omega \left(t\right)$, as defined in [14]. We validate our ANNs controller based on FA with SP on the quadrotor, ensuring it tracks the reference trajectory within a MATLAB/Simulink simulation environment. The parameters of this section are selected as shown in Table 1; all simulations are performed with continuous-time integration, and using a numerical step of dt=0.001 s. The inertia matrix is $J=\left[\begin{array}{ccc} 0.00065 & 0 & 0\\ 0 & 0.00065 & 0\\ 0 & 0 & 0.0013 \end{array}\right]\;\mathrm{kg}\cdot m^{2}$.

**Remark** **5.** *The selection of controller parameters was guided by both theoretical stability conditions (see Section 4) and empirical validation. Parameters such as learning rate and regularization were chosen to guarantee closed-loop stability, while final values were fine-tuned for best performance in simulation and experiments.*

**Figure 4:**
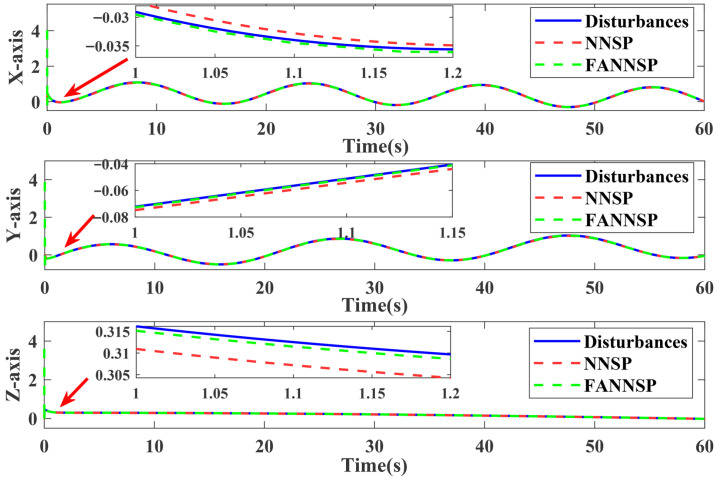
Comparison of the performance with NNSP and FANNSP in approximating the unknown terms $\Delta_{d}$.

**Figure 5:**
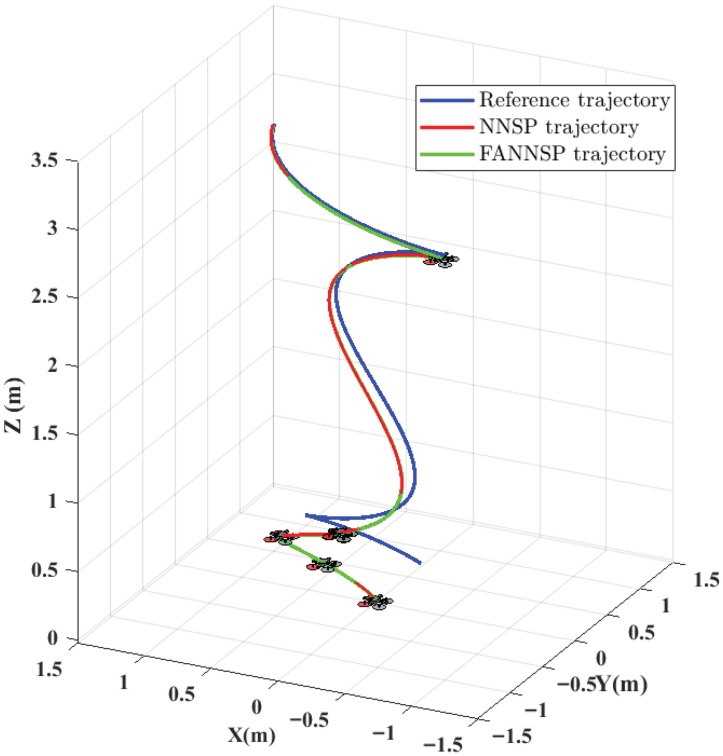
Trajectories of the quadrotor under different controllers.

**Figure 6:**
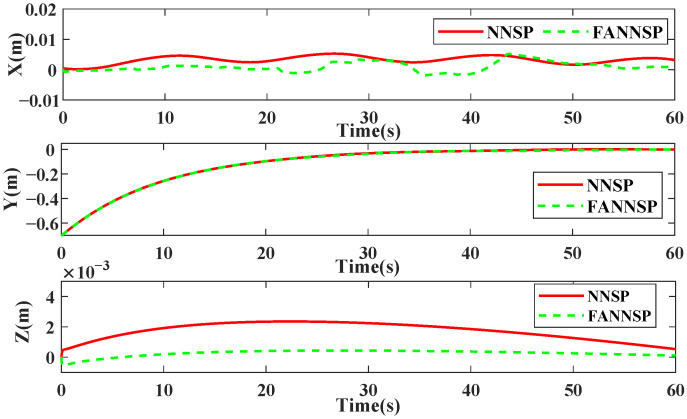
Trajectory errors of the quadrotor with NNSP and FANNSP, respectively.

**Figure 7:**
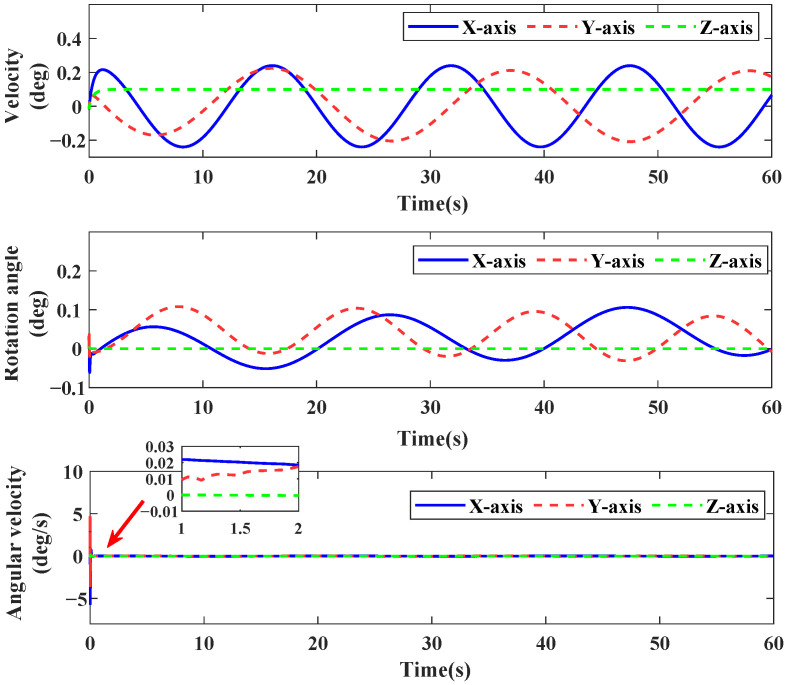
Output signals of the quadrotor with FANNSP.

Figure 4 illustrates the approximation and compensation capabilities of NNSP (RBF NNs with SP) and FANNSP (ANN based on FA with SP) in the presence of unknown internal and external disturbance terms $\Delta_{d}$. Compared to NNSP, FANNSP exhibits superior approximation and compensation capabilities for the unknown terms $\Delta_{d}$ with a higher approximation speed. By combining Figure 5 and Figure 6, it can be observed that the quadrotor demonstrates better trajectory tracking with FANNSP compared to NNSP, and the tracking errors converge more quickly from the same initial position. For the quadrotor, Figure 7 shows the output signals for velocity, rotation angle, and angular velocity, respectively, all within a reasonable value range when using FANNSP.

## 6. Experimental Results

**Figure 8:**
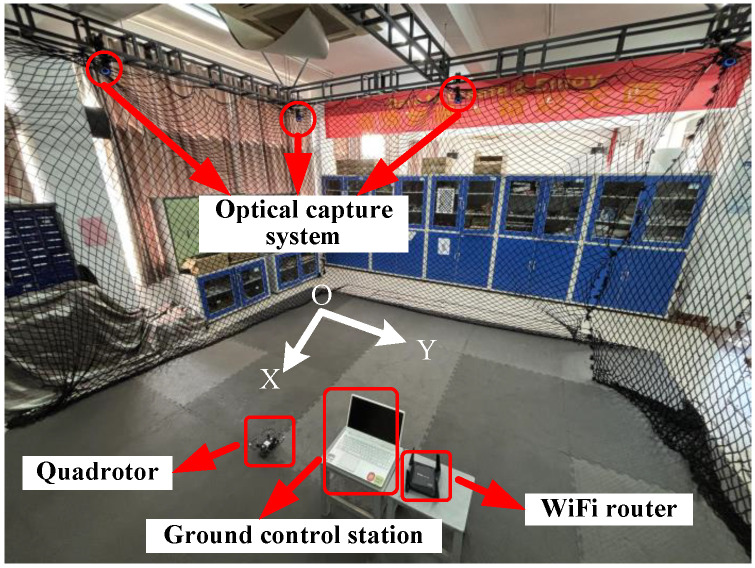
Experimental environment.

To further illustrate the practical application of our designed FANNSP for quadrotors in engineering, we validate FANNSP in the experimental environment shown in Figure 8 and compare the experimental results with the traditional PID controller and NNSP. In Figure 8, the quadrotor, with dimensions of 200 × 200 × 85 (mm) and a weight of 310 (g), communicates with the control station via WiFi and is positioned by an optical capture system. This system consists of 12 OptiTrack Prime 13 cameras, each with a resolution of 1280×1024 and a frame rate of 240 FPS. To accommodate our experimental environment, we incorporate a hysteresis quantizer [23] to reduce the signal transmission burden and quantify the control signals for the quadrotor.

We considered two different external disturbances: ground effect and continuous wind from a fan. In these two scenarios, we examined and compared the control efficacy of the traditional PID controller, NNSPQ (RBF NNs with SP and hysteresis quantizer) and FANNSPQ (ANN based on FA with SP and hysteresis quantizer) on the quadrotor. The coefficients of PID for this experimental part are as follows: $K_{P}=$ diag{2.5,2.5,0.7}, $K_{I}=$ diag{0.000002,0.000002,0.002}, $K_{D}=$ diag{0.000002,0.000002,0.02}.

**Remark** **6.** *Unlike many existing studies that validate controllers through simulations alone, we provide real-world experimental validation of our approach on a quadrotor platform. The experiments are conducted under two distinct disturbance scenarios: ground effect and continuous wind disturbances. Our results show that the ANN controller with FA and SP outperforms traditional PID control and RBF NN with SP in both trajectory tracking accuracy and stability. The real-world results confirm the effectiveness of our approach, demonstrating that it performs better than existing methods in dynamic, real-world conditions.*

### 6.1. Case 1

In this case, we primarily utilize the ground effect as the disturbance, as discussed in [1,2]. We set the UAV flying height to 0.25 m and define the reference trajectory as p = ${\left[1.5\sin\left(0.4t-1/2\pi \right),0.5\sin\left(0.8t\right),0.25\right]}^{T}$ (m).

**Figure 9:**
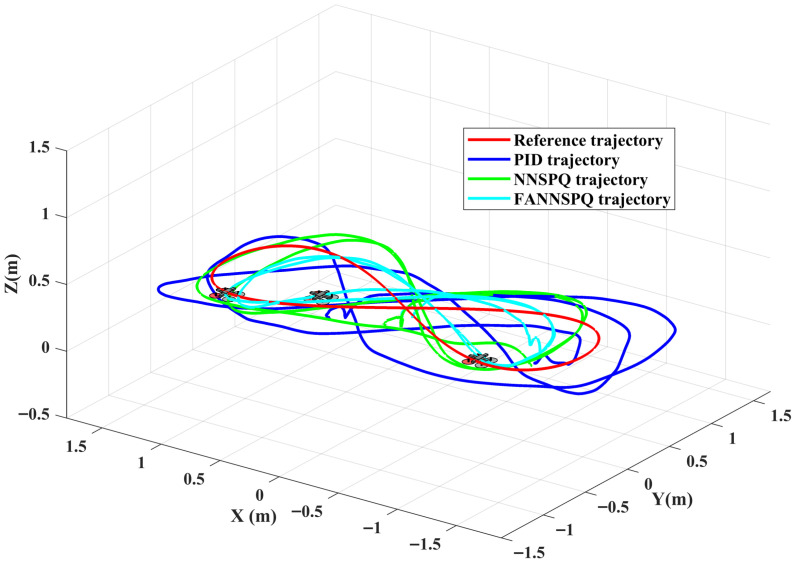
Experimental trajectories of the quadrotor in different controllers with the ground effect in three dimensions.

**Figure 10:**
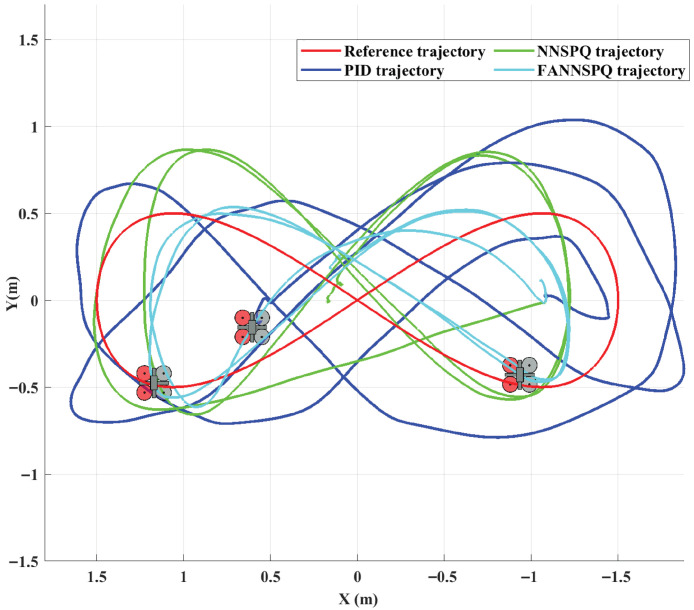
Experimental trajectories of the quadrotor in different controllers with the ground effect in two dimensions.

**Figure 11:**
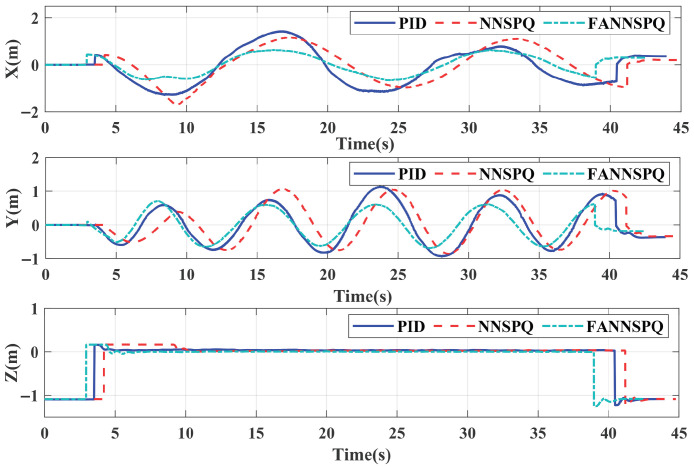
The errors of trajectories in different controllers with the ground effect.

**Figure 12:**
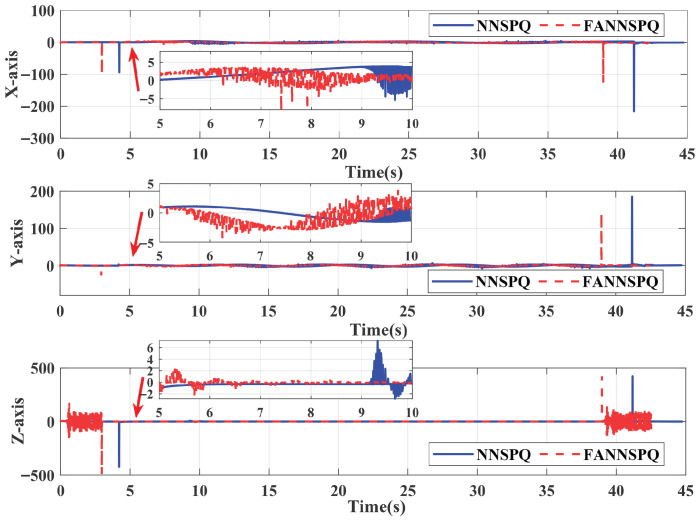
The input signals of NNSPQ and FANNSPQ for the quadrotor in Case 1.

Figure 9, Figure 10 and Figure 11 demonstrate that PID, NNSPQ and FANNSPQ enable the quadrotor to reach the predetermined altitude. Compared to PID, as described in [7], both NNSPQ and FANNSPQ allow the quadrotor to track the desired trajectory more quickly and exhibit effective trajectory error convergence. Furthermore, compared to NNSPQ, FANNSPQ provides a more stable flight with smaller trajectory errors during the actual quadrotor flight. Additionally, with the hysteresis quantizer, Figure 12 shows that the control signals of both NNSPQ and FANNSPQ for the quadrotor are well-quantized, where the observed peak values are mainly caused by transient switching across quantization thresholds during the initial response.

### 6.2. Case 2

In this case, we consider the external disturbance caused by continuous wind from a fan. The quadrotor is set to fly at an altitude of 1 m, and the reference trajectory is defined as p = ${\left[1.5\sin\left(0.4t-1/2\pi \right),0.5\sin\left(0.8t\right),1\right]}^{T}$ (m). Similar to Case 1, we examine and compare the control efficacy of PID, NNSPQ, and FANNSPQ on the quadrotor.

**Figure 13:**
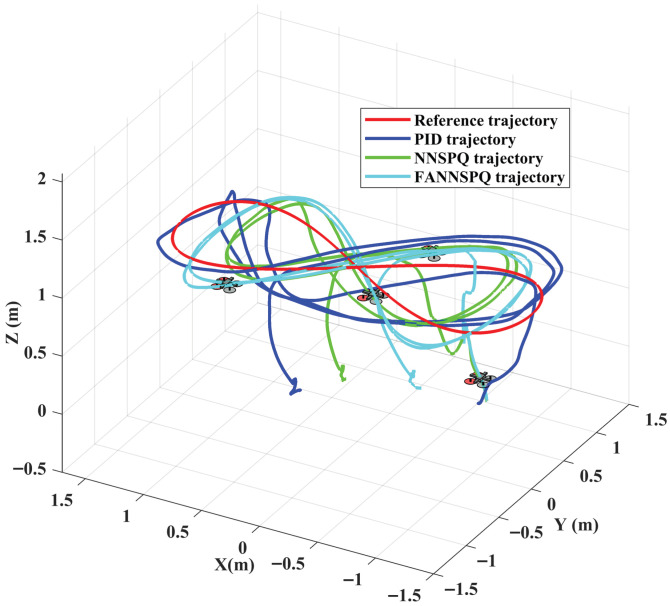
Experimental trajectories of the quadrotor in different controllers with the external continuous disturbance from a fan in three dimensions.

**Figure 14:**
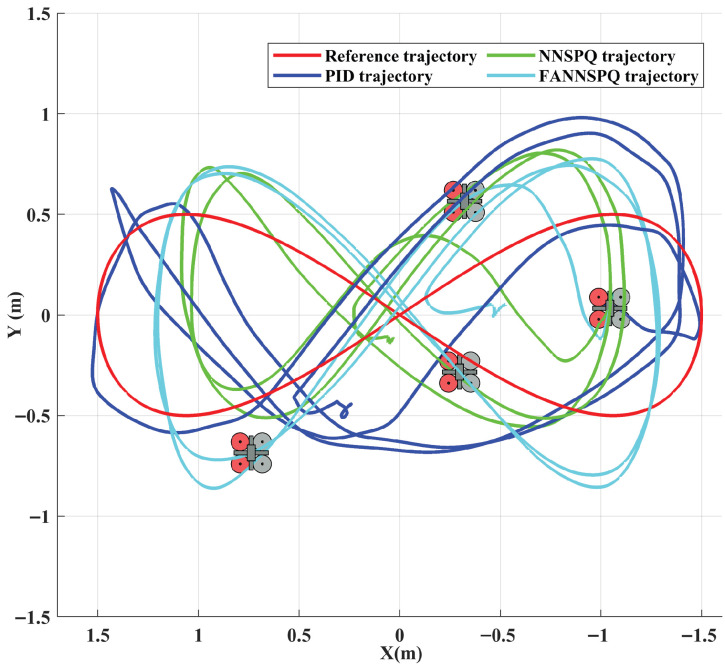
Experimental trajectories of the quadrotor in different controllers with the external continuous disturbance from a fan in two dimensions.

**Figure 15:**
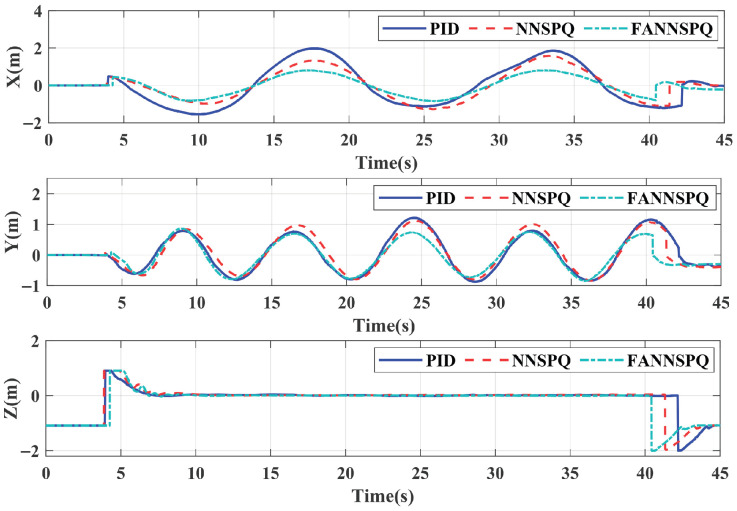
The errors of trajectories in different controllers with the external continuous disturbance from a fan.

**Figure 16:**
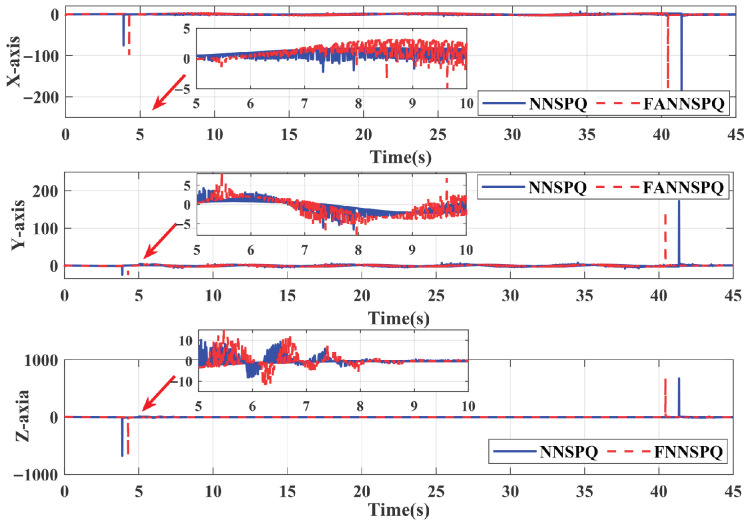
The input signals of NNSPQ and FANNSPQ for the quadrotor in Case 2.

By combining Figure 13, Figure 14 and Figure 15, it is evident that PID, NNSPQ and FANNSPQ all enable the quadrotor to reach the predetermined altitude. However, the impact of the fan in Case 2 is greater than the ground effect in Case 1. Compared with PID, both NNSPQ and FANNSPQ enable the quadrotor to track the desired trajectory more quickly, demonstrating effective trajectory error convergence. Furthermore, FANNSPQ provides a more stable flight and smaller trajectory errors compared to NNSPQ. Additionally, incorporating the quantizer, which can reduce the transmission burden, results in well-quantized control signals for NNSPQ and FANNSPQ, as shown in Figure 16, with the peak magnitudes reflecting transient control efforts rather than steady-state behavior.

## 7. Conclusions

This article designs an ANN controller based on the FA to address the challenges posed by the unknown internal and external disturbance terms, ensuring efficient tracking of the quadrotor along the reference trajectory. FA is designed and incorporated into the structure of ANN, which enhances the characterization of input signal data and can improve the learning accuracy of NN in approximating the unknown terms. Additionally, an SP is designed to improve the ANN’s approximation speed to the unknown disturbance terms. Through stability analysis, the closed-loop system of the quadrotor is proved to be ISS. The simulation results demonstrate that ANN based on FA with SP effectively estimate the unknown disturbance terms compared to NNSP. With two different experimental scenarios, ground effects and continuous wind from a fan, we observe that our controller design, FANNSPQ, makes the error converge quickly and improves the effectiveness of the quadrotor’s trajectory tracking in real-word conditions compared to the traditional PID controller and NNSPQ. Future research will focus on optimizing the network structure, improving robustness to system parameter variations and communication issues, and providing stronger global convergence guarantees.

## Figures and Tables

**Table 1:** The parameters of quadrotor in the simulation.

Parameters	Symbols and Values
	$K_{p}$ = diag{0.1,0.1,0.1};
Error	$K_{v}$ = diag{20,20,20};
Coefficients	$K_{\Theta }$ = diag{15,15,15};
	$K_{\omega }$ = diag{20,20,20}.
Quadrotor	m=0.2 (kg);
Parameters	g=9.81.
FANNSP	$K_{FA}$ = diag{200,200,200};
Parameters	η = 10,000; σ=0.0001.
The Unknown	$\Delta_{d}=[-2.5\sin\left(v_{x}\right)+0.5\cos\left(0.02t\right);$
Disturbance	$-3\sin\left(v_{y}\right)+0.5\sin\left(0.02t\right);$
Terms	$-2\sin\left(v_{z}\right)+0.5\cos\left(0.02t\right)]$.

## Data Availability

The data that support the findings of this study are available from the corresponding author upon reasonable request.
